# Habenula Connectivity and Intravenous Ketamine in Treatment-Resistant Depression

**DOI:** 10.1093/ijnp/pyaa089

**Published:** 2020-11-29

**Authors:** Ana Maria Rivas-Grajales, Ramiro Salas, Meghan E Robinson, Karen Qi, James W Murrough, Sanjay J Mathew

**Affiliations:** 1 Menninger Department of Psychiatry and Behavioral Sciences, Baylor College of Medicine, Houston, Texas, USA; 2 Department of Neuroscience, Baylor College of Medicine, Houston, Texas, USA; 3 Mental Health Care Line, Michael E. DeBakey VA Medical Center, Houston, Texas, USA; 4 The Menninger Clinic, Houston, Texas, USA; 5 Core for Advanced Magnetic Resonance Imaging and Department of Neurosurgery, Baylor College of Medicine, Houston, Texas, USA; 6 Department of Cognitive Neuroscience, Rice University, Houston, Texas, USA; 7 Depression and Anxiety Center for Discovery and Treatment, Department of Psychiatry; Department of Neuroscience; and Friedman Brain Institute, Icahn School of Medicine at Mount Sinai, New York, New Yorks, USA

**Keywords:** Habenula, ketamine, resting-state functional MRI, treatment-resistant depression

## Abstract

**Background:**

Ketamine’s potent and rapid antidepressant properties have shown great promise to treat severe forms of major depressive disorder (MDD). A recently hypothesized antidepressant mechanism of action of ketamine is the inhibition of N-methyl-D-aspartate receptor–dependent bursting activity of the habenula (Hb), a small brain structure that modulates reward and affective states.

**Methods:**

Resting-state functional magnetic resonance imaging was conducted in 35 patients with MDD at baseline and 24 hours following treatment with i.v. ketamine. A seed-to-voxel functional connectivity (FC) analysis was performed with the Hb as a seed-of-interest. Pre-post changes in FC and the associations between changes in FC of the Hb and depressive symptom severity were examined.

**Results:**

A reduction in Montgomery–Åsberg Depression Rating Scale scores from baseline to 24 hours after ketamine infusion was associated with increased FC between the right Hb and a cluster in the right frontal pole (*t *= 4.65, *P *= .03, false discovery rate [FDR]-corrected). A reduction in Quick Inventory of Depressive Symptomatology-Self Report score following ketamine was associated with increased FC between the right Hb and clusters in the right occipital pole (*t* = 5.18, *P* < .0001, FDR-corrected), right temporal pole (*t *= 4.97, *P* < .0001, FDR-corrected), right parahippocampal gyrus (*t *= 5.80, *P* = .001, FDR-corrected), and left lateral occipital cortex (*t *= 4.73, *P* = .03, FDR-corrected). Given the small size of the Hb, it is possible that peri-habenular regions contributed to the results.

**Conclusions:**

These preliminary results suggest that the Hb might be involved in ketamine’s antidepressant action in patients with MDD, although these findings are limited by the lack of a control group.

**Significance Statement**

The habenula, a small brain structure involved in reward and affective states, has been implicated in the rapid antidepressant effect of ketamine. In the present study, we used resting-state functional magnetic resonance image (fMRI) to evaluate connectivity changes of the habenula 24 hours after a single infusion of ketamine in a group of patients with treatment-resistant depression. This study shows that ketamine’s rapid antidepressant effects are associated with changes in the connectivity between the habenula and brain structures important in mood regulation, and provides preliminary clinical evidence of the involvement of the habenula in ketamine’s mechanism of action.

## Introduction

Major depressive disorder (MDD) is a chronic and disabling illness that affects almost one-quarter of the world’s population (World Health Organization, 2004; Whiteford et al., 2013). A large proportion of patients do not respond adequately to conventional treatments despite multiple trials of monoaminergic-based antidepressants and augmentation strategies (Rush et al., 2006). Furthermore, these medications usually take several weeks to months to achieve an antidepressant response or remission. The discovery of the rapid antidepressant effect of ketamine has opened an avenue for new drug discovery in depression. Ketamine’s rapid antidepressant effect in patients with treatment-resistant depression (TRD) is observed within several hours of a single subanesthetic i.v. infusion, with response rates of 50–60% over the first 3 days post-infusion (Zarate et al., 2006; Murrough et al., 2013; Fava et al., 2020). This rapid and robust response offers a unique paradigm to explore the neurobiology of depression and the mechanism of action of rapidly acting antidepressants (Abdallah et al., 2018).

Ketamine’s pharmacological activity includes modulation of glutamate neurotransmission via antagonism of the N-methyl-D-aspartate receptor. Abnormalities in glutamate signaling have been implicated in the pathophysiology of depression (Duman and Aghajanian, 2012; Murrough et al., 2017). Ketamine is hypothesized to improve depression by restoring network dynamics in glutamatergic synapses and circuits that mediate stress resilience and mood regulation (Zanos and Gould, 2018). From a neural-circuit perspective, some authors propose that the rapid antidepressant effect of ketamine is mediated by a small number of nodes or a single node (Abdallah et al., 2018; Zanos and Gould, 2018). Based on the observations that ketamine has a short elimination half-life (approximately 3 hours in humans) (Clements et al., 1982), it has been hypothesized that this node (or nodes) should have NMDA channels intrinsically open. Also, because ketamine can rapidly elevate the levels of dopamine, serotonin, and norepinephrine, the brain areas mediating the antidepressant effects of ketamine may suppress nuclei in the midbrain where these neurotransmitters originate (Cui et al., 2019). The habenula (Hb), which provides top-down regulation to monoaminergic centers in the midbrain, has recently emerged as a promising area of investigation.

The Hb is a diencephalic structure that acts as an interface between the limbic forebrain and brainstem nuclei that modulate reward and affective states (Salas et al., 2010; Namboodiri et al., 2016). Unlike neurons in the brain’s mesolimbic system that promote reward-seeking behavior, neurons in the Hb encode information related to aversive outcomes and promote behavioral avoidance (Matsumoto and Hikosaka, 2007). The lateral Hb (LHb) specifically can inhibit reward-related dopaminergic neurons in the ventral tegmental area via the rostromedial tegmental nucleus, which sends GABAergic projections to the ventral tegmental area (VTA) and feedforward inhibition to monoaminergic nuclei in the midbrain (Namboodiri et al., 2016). Evidence from preclinical studies demonstrates that LHb neurons are hyperactive and have increased synaptic excitability in rodent models of depression (Li et al., 2011; Park et al., 2017). In human neuroimaging studies using task-based fMRI, depressed patients have displayed greater Hb activation to noxious stimuli (Roiser et al., 2009; Lawson et al., 2017) compared with healthy volunteers, while deep brain stimulation to this structure improved depressive symptoms in a case of TRD (Sartorius et al., 2010). In addition, Hb connectivity, as measured with resting-state fMRI, can be used as a predictor of antidepressant response (Gosnell et al., 2019). More recently it was shown that local infusion of ketamine in the LHb blocks burst-firing activity and induces antidepressant-like responses in an animal model of depression (Shepard et al., 2018; Yang et al., 2018) and that ketamine-induced Hb changes are modulated by opioid receptors (Klein et al., 2020).

Structural and functional imaging data in depressed patients indicate that ketamine induces changes in brain areas related to reward and mood regulation, such as the anterior cingulate cortex, caudate, hippocampus, and amygdala (Murrough et al., 2015; Abdallah et al., 2017; Ionescu et al., 2018). However, apart from 1 fluorodeoxyglucose-positron emission tomography study that reported reduced metabolism in the right Hb following ketamine treatment (Carlson et al., 2013), there are no previous human neuroimaging studies to our knowledge evaluating the possible role of the Hb in ketamine’s antidepressant effect. In this study, we used resting-state fMRI to examine possible changes in functional connectivity (FC) of the Hb in patients with TRD who received a single subanesthetic dose infusion of ketamine. Based on the observations that ketamine enhances the connectivity between frontal and limbic structures and that clinical improvement in depression is associated with better cognitive control of emotions via prefrontal cortical regions (Johnstone et al., 2007; Murrough et al., 2011), we hypothesized that improvement of depression severity would be associated with greater connectivity between the Hb and prefrontal areas. Also, given that the Hb inhibits reward-related regions in the brainstem (Namboodiri et al., 2016) and that it has been observed that ketamine acts by reducing the tonic firing of the Hb (Yang et al., 2018), we hypothesized that improvement of depressive symptoms would be associated with a reduced connectivity of the Hb and the VTA.

## Methods

### Participants

This study included 35 individuals with MDD and a history of nonresponse to at least 2 antidepressant trials who were recruited from Baylor College of Medicine (site 1, n* *= 9) and Icahn School of Medicine at Mount Sinai (site 2, n* *= 26). Enrollment and study procedures for the parent clinical trials have been previously described at clinicaltrials.gov (NCT00768430, NCT01880593). Patients (21–80 years of age) were included if they were antidepressant or antipsychotic free for at least 1 week prior to imaging (as needed, benzodiazepines were allowed but withheld the day of each scan) and met the criteria for current major depressive episode as determined by the Structured Clinical Interview for DSM-IV-Patient Edition (First et al., 1995). Participants were excluded for any of the following: a lifetime history of a psychotic illness or bipolar disorder, alcohol or substance abuse in the previous 2 years, unstable medical illness, serious and imminent suicidal or homicidal risk, and an MRI contraindication. The study was approved by the local institutional review boards. Participants gave their informed consent and were compensated for their time.

### Study Procedures

Following medical and psychiatric assessments, patients were randomly assigned in a 2:1 ratio to receive a single i.v. infusion of ketamine hydrochloride (0.5 mg/kg) or midazolam (0.045 mg/kg) infused over 40 minutes. Due to low numbers of patients who received midazolam and were scanned (n = 9), only patients who received ketamine (n = 35) are reported here. Patients underwent MRI and clinical assessments at 2 time points: baseline, at least 24 hours prior to infusion (Time 1), and 24 hours following a single i.v. infusion of ketamine or midazolam (Time 2). Depression severity was measured using the Montgomery–Åsberg Depression Rating Scale (MADRS) (Montgomery and Åsberg, 1979) and the Quick Inventory of Depressive Symptomatology-Self Report (QIDS-SR) (Rush et al., 2003) at baseline and 24 hours post treatment.

### Imaging Acquisition and Preprocessing

At site 1, images were obtained on a 3-Telsa Siemens Trio Magnetom system. Acquisition parameters for the functional images were repetition time (TR): 2000 ms, echo time (TE): 30 milliseconds, flip angle: 90, voxel size: 3.4 mm × 3.4 mm × 4mm, and field of view: 220 mm × 220 mm. For co-registration, structural T1 images were acquired using a Magnetization-Prepared Rapid Acquisition Gradient Echo with the following parameters: TR (outer loop): 2500 milliseconds, TE: 2.92 milliseconds, flip angle: 12, voxel size: 1 mm × 1 mm × 1 mm, field of view: 245 mm × 245 mm, 160 slices. At site 2, images were acquired on a 3-Telsa Philips Achieva system. Functional images were acquired with these parameters: TR: 2000 milliseconds, TE: 27 milliseconds, flip angle: 90, voxel size: 2.18 mm × 2.18 mm × 2.18 mm, field of view: 210 mm × 210 mm. Acquisition parameters for the Magnetization-Prepared Rapid Acquisition Gradient Echo structural data were TR (inner loop): 7.5 milliseconds, TE: 3.5 milliseconds, flip angle: 8, voxel size: 1 mm × 1 mm × 1 mm, field of view: 224 mm × 224 mm, 172 slices. “Site” was entered as a covariate in second-level analysis to account for differences in acquisition parameters across sites.

Preprocessing was performed using the CONN Functional Connectivity toolbox Version 18b (http://www.nitrc.org/projects/conn) within SPM12 (http://www.fil.ion.ucl.ac.uk/spm/). For each participant, functional images were realigned, co-registered with each participant’s anatomical image, normalized to the Montreal Neurological Institute template, and smoothed using an 8-mm Gaussian kernel. When performing seed-to-voxel FC in CONN, the seed is by default not smoothed. Thus, we smoothed only the resulting clusters but not the Hb. Anatomical landmarks in the normalized anatomical and functional data were visually checked and compared against the Montreal Neurological Institute template for each participant. The Artifact Detection Toolbox (http://www.nitrc.org/projects/artifact_detect/) was used to repair artifac due to frame-by-frame head movement and correct global drift. Motion outliers were defined as volumes that exceeded 5 z-normalized SDs away from the mean global brain signal across the entire volume or a composite movement threshold of 0.9 mm scan-to-scan frame-wise displacement. The anatomical component correction method (Behzadi et al., 2007) was used to remove physiological noise from white matter and cerebrospinal fluid (CSF), as implemented in CONN. Motion outliers, realignment parameters, and white matter and CSF masks were included as covariates and regressed out of the blood-oxygen-level-dependent signal before computing connectivity measures. Data were band-pass filtered between 0.008 and 0.09 Hz to focus on low-frequency correlations. As mentioned, the Hb seeds were not smoothed to decrease likely contamination with adjacent areas.

### FC Analysis

**Figure 1.**
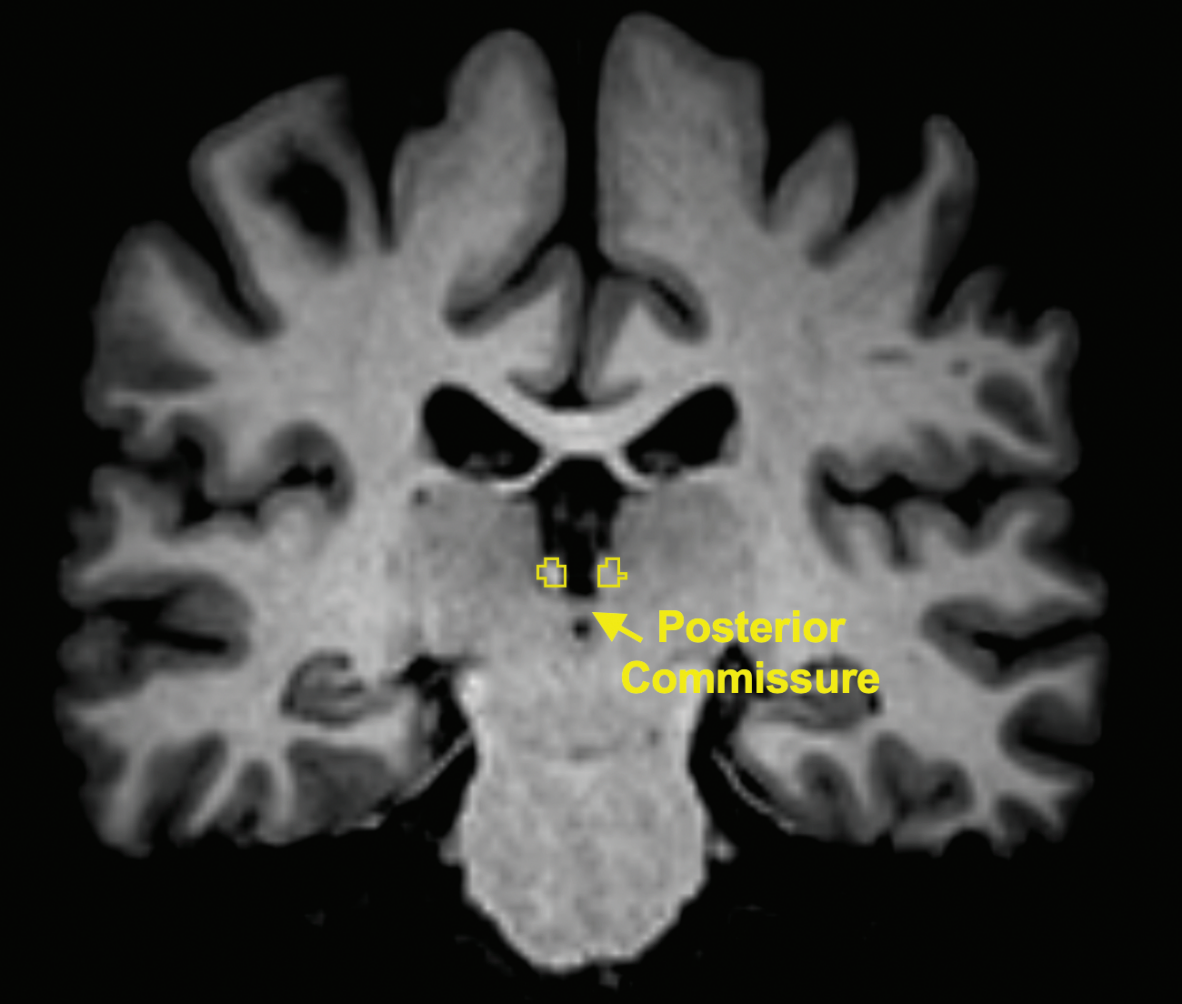
Delineation of the habenula on a T1-weighted coronal image.

**Figure 2.**
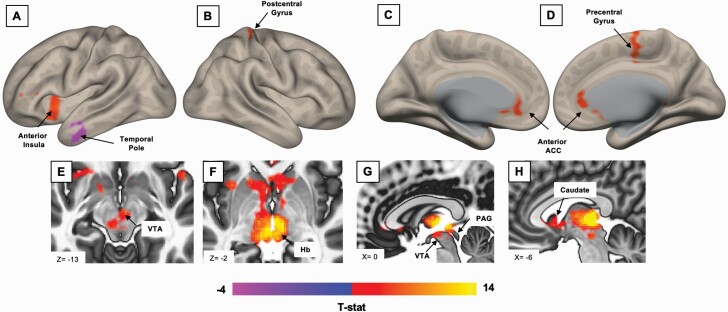
Whole-brain resting-state Hb connectivity maps for significant (voxel-wise threshold naive *P* < .01, cluster-wise threshold *P* < .05) findings for all participants on a semi-inflated cortical surface (A–D) and in subcortical MNI space (E–H). Abbreviations: PAG, periaqueductal grey matter; VTA, ventral tegmental area.

A seed-to-voxel connectivity analysis was conducted with the right and left Hb as seeds-of-interest. Individual specific Hb seeds were manually created on the normalized structural images using 3D Slicer Version 4.5 (https://www.slicer.org). The Hb is located anterior to the pineal gland and occupies the same z-coordinate of the posterior commissure, which can be easily identified in coronal slides (Figure 1). Dorsal to the posterior commissure, the Hb can be seen protruding into the third ventricle in both sides of the midsagittal plane. We used a variation of the method described by Lawson et al. (2013) to identify the posterior, lateral, and anterior boundaries of the Hb. A 2-mm-radius sphere was centered within these boundaries in left and right Hb of each participant defined in the anatomical image and was manually inspected to ensure that CSF was not included in these voxels. Additionally, we included regressors from 3-mm spheres located 6 mm lateral to the left and right Hb seeds (in template space) to rule out contamination from nearby thalamic areas. To validate the placement of these region of interest, we compared our Hb connectivity findings at baseline with those reported in Ely et al. (2016, 2019), Torrisi et al. (2017), and Curtis et al. (2017). As reported in these studies, we observed that the Hb was highly positively correlated with the thalamus, caudate, anterior and posterior cingulate, and insula and negatively correlated with areas in the temporal and occipital lobes (Figure 2). We compared Pearson correlation coefficients between the mean signal time course between the Hb seeds and the whole brain. Based on previous evidence suggesting different connectivity patterns between the right and left Hb (Hétu et al., 2016; Gosnell et al., 2019; Luan et al., 2019), these were evaluated separately. Correlation coefficients were then normalized using Fisher’s r-to-z transformation and implemented in a general linear model.

### Statistical Analysis

Our primary interest was to investigate the associations between improvement of depression severity and changes in the FC of the Hb after ketamine treatment. To investigate time-related changes in the FC of the Hb, we conducted a paired-sample *t* test between the scan at Time 1 (pre-treatment baseline) and the scan at Time 2 (24 hours post-treatment). Clinical improvement (difference in MADRS and QUIDS-SR scores between Time 1 and Time 2) was used as a covariate of interest. “Site” was used as nuisance covariate. Between-condition contrasts were defined as Time 2 > Time 1. Results were thresholded at voxel level *P* < .001 (height threshold) and then corrected for whole-brain comparisons using the False Discovery Rate correction for whole-brain comparisons at *P* < .05 (extent threshold) (Friston et al., 1994). We used general linear modeling to examine MADRS scores at 24 hours as a function of treatment after controlling for site.

## Results

Demographic and clinical data are shown in Table 1. Twenty-four hours following ketamine treatment, 20 patients (57%) achieved response (defined as a reduction in the baseline MADRS score by 50% or more), and 14 patients (40%) showed remission of symptoms (defined as a MADRS score ≤ 9 post treatment). There was a significant main effect of ketamine on depression severity (*F *= 98.0, df = 1,33, *P* < .001). MADRS scores at 24 hours did not differ as a function of site (F = 0.004, df = 1,32, *P* = .95).

**Table 1.** Demographic and clinical characteristics

Age (y)	42.2 ± 13.9
Gender (male/female)	19/16
Education (y)	16 ± 3
Age of onset (y)	18.3 ± 8.6
No. of antidepressant trials	4.0 ± 3.0
Duration of current episode	
Acute (<12 mo), n (%)	7 (20%)
Subacute (13–24 mo), n (%)	6 (17%)
Chronic (>25 mo), n (%)	21 (60%)
Hospitalizations for MDD, n (%)	13 (37%)
History of suicide attempt, n (%)	9 (25%)
Comorbid anxiety disorder, n (%)	13 (37%)
Past ETOH abuse, n (%)	5 (14%)
Baseline MADRS	30.6 ± 5.2
Post treatment MADRS	13.7 ± 9.4
Baseline QIDS-SR	16.2 ± 4.4
Post treatment QIDS-SR	8.5 ± 6.4

Abbreviations: ETOH, alcohol; MADRS, Montgomery–Åsberg Depression Rating Scale; QIDS-SR, Quick Inventory of Depressive Symptomatology-Self Report.

Values are means and SD.

### Hb Connectivity Changes Following Ketamine

**Figure 3.**
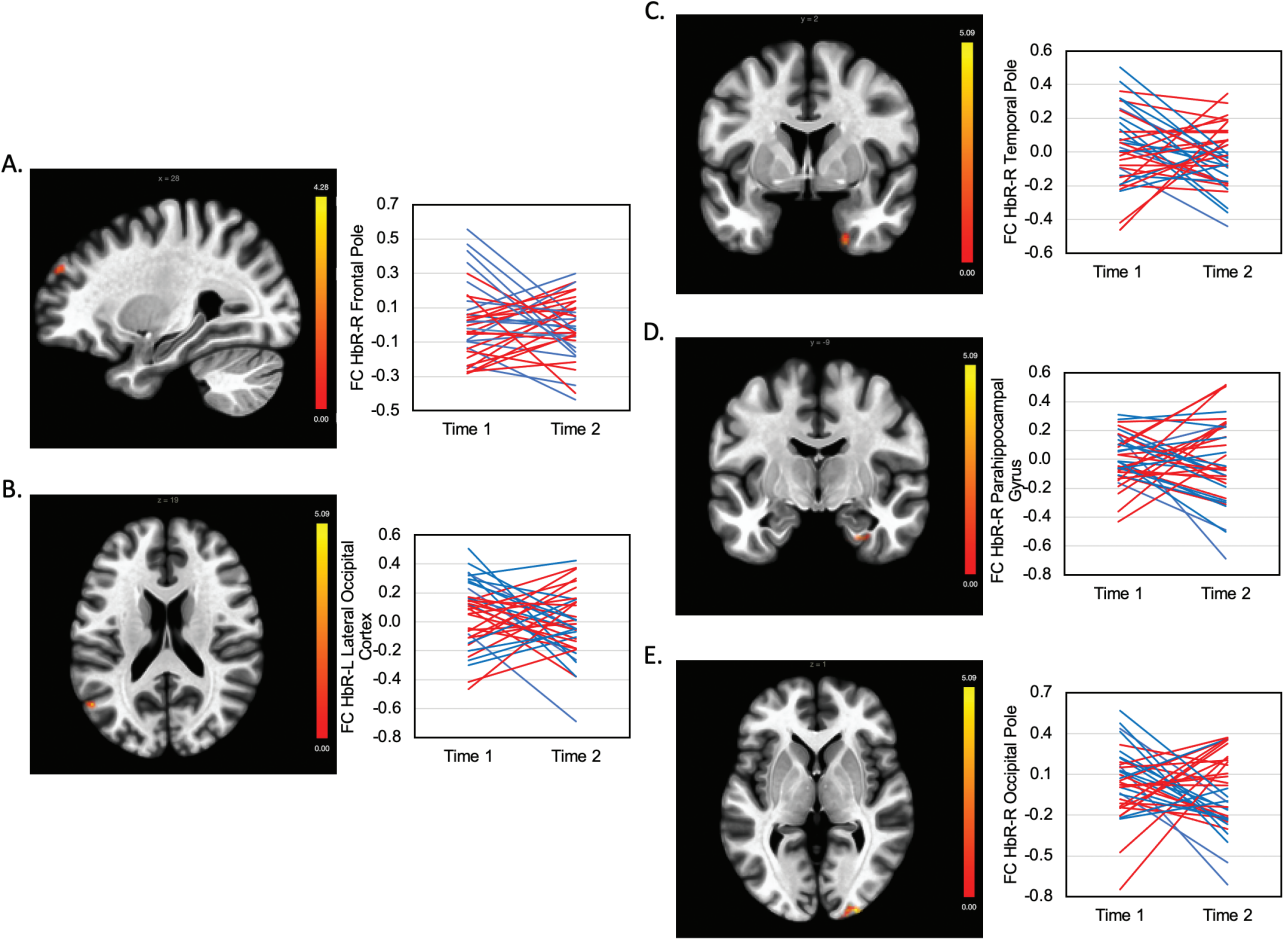
Functional connectivity (FC) of the habenula (Hb) in patients treated with ketamine. The plots show changes in FC between the right Hb and significant clusters at Time 1 (pre-ketamine) and Time 2 (post ketamine). The red lines indicate patients with higher improvement in depression severity, and blue lines indicate patients with lower improvement based on a median split of the changes in Montgomery–Åsberg Depression Rating Scale (MADRS) score and Quick Inventory of Depressive Symptomatology-Self Report score (QIDS-SR). (A) Cluster in which an increase of FC between the right Hb and the right frontal pole significantly correlated with a reduction in MADRS score. (B–E). Clusters in which an increase of FC between the right Hb and the left lateral occipital pole (B), right temporal pole (C), right parahippocampal cortex (D), and right occipital pole (E) significantly correlated with a reduction in QIDS-SR score.

There was a main effect of time in the FC between the right Hb and a cluster in the left hippocampus and left parahippocampal gyrus (*t *= 5.93, *P* = .001). A reduction of MADRS scores from Time 1 (baseline) to Time 2 (24 hours after infusion) was associated with an increase in FC between the right Hb and a cluster in the right frontal pole ( *t* = 4.65, *P* = .03) (Table 2; Figure 3A). There were no other associations between changes in FC in the right or left Hb and changes in MADRS scores. A reduction in QIDS-SR scores was associated with increased FC between the right Hb and clusters in the right occipital pole (*t* = 5.18, *P* < .0001) (Figure 3B), right temporal pole (t = 4.97, *P* < .0001) (Figure 3C), right parahippocampal gyrus (t = 5.80, *P* = .001) (Figure 3D), and left lateral occipital cortex (t = 4.73, *P* = .03) (Figure 3E).

**Table 2.** Association between improvement in depression severity and FC changes in the right Hb

Region	Side	Cluster size (voxels)	*P*-FDR	MNI peak coordinates
MADRS				
Frontal pole (BA 9)	R	69	0.03	+30 + 50 + 32
QIDS-SR				
Occipital pole (BA 18)	R	197	0.00014	+28 −98 + 02
Temporal pole (BA 38)	R	114	0.00794	+22 + 12 −44
Parahippocampal cortex (PHC)	R	100	0.001	+12 −12 −34
Lateral occipital cortex (BA 39)	L	51	0.03	−56 −64 + 22

Abbreviations: BA, Broadman area; FDR, false discovery rate; L, left; MADRS, Montgomery–Åsberg Depression Rating Scale; MNI, Montreal Neurosciences Institute; QIDS-SR, Quick Inventory of Depressive Symptomatology-Self Report; R, right.

## Discussion

The Hb has been implicated in the rapid antidepressant effect of ketamine (Yang et al., 2018). In the present study, we used resting-state fMRI to evaluate the changes in the FC of the Hb 24 hours after a single infusion of i.v. ketamine in a group of patients with TRD. While some of our results were consistent with our original hypothesis that ketamine’s rapid antidepressant effects would be associated with increased FC between the Hb with prefrontal areas, other results were less consistent. We expected but did not observe any associations with limbic areas that are known to be anatomically connected to the Hb, such as the VTA, striatum, and medial prefrontal cortex (PFC) (Namboodiri et al., 2016; Hu et al., 2020). As may be expected due to the challenges of conducting imaging studies on the Hb (e.g., small size and location near sources of physiological noise), the identified clusters were small and had only modest effects. Due to the small size of the Hb, even with intense changes, we would expect only a few voxels to be affected, resulting in small clusters. Further, the Hb is located next to the ventricles, which contribute significant physiological noise to our region of interest through blurring and partial volume effects, and this may have reduced the significance of the finding. Furthermore, there was little overlap between the clusters associated with improvement as measured with the MADRS vs the QIDS-SR. However, the locations of these clusters were in good agreement with the literature describing the expected effects of ketamine on the brain (Carlson et al., 2013; Downey et al., 2016; Abdallah et al., 2017), providing confidence in our results.

Abnormalities in glutamatergic neurotransmission has been proposed as an underlying neural correlate of depression (Abdallah et al., 2015; Murrough et al., 2017). Based on this model, ketamine exerts its antidepressant effects by restoring network abnormalities in regions important in stress, resilience, and mood regulation (Abdallah et al., 2015). Specifically, it has been proposed that ketamine has an ability to improve the cognitive control of emotions by enhancing the connectivity between frontal regions to deeper limbic structures, resulting in an improvement of depressive symptoms (Ionescu et al., 2018). The finding of increased FC between the Hb and the cluster in the frontal pole, which is part of the dorsolateral PFC, is consistent with previous reports showing enhanced connectivity within and to frontal structures after ketamine treatment. For example, Abdallah et al. (2017) showed that ketamine responders had increased global brain connectivity in the lateral PFC, caudate, and insula compared with nonresponders. In another functional MRI study, Downey et al. (2016) showed increased blood-oxygen-level-dependent signal in the subgenual PFC associated with antidepressant response in patients that received ketamine. More recently, Gärtner et al. (2019) showed that antidepressant response to ketamine was associated with increased connectivity between the subgenual cingulate and PFC.

The finding of increased FC in the parahippocampal cortex (PHC) is consistent with previous reports showing a role of the PHC in depression (Carlson et al., 2013; Harel et al., 2016; Karim et al., 2018; Ambrosi et al., 2019). The PHC is part of a brain network that mediates contextual associative processing (Aminoff et al., 2013). This network is active when individuals are presented with images that are strongly associated with a specific context compared with those that are not. Previous research suggests a link between mood and associative thinking by which negative mood narrows while positive mood expands the amount of associations (Isen et al., 1987). Abnormalities in PHC function and connectivity in depressed patients have also been reported. For example, Harel et al. (2016) showed that depressed patients exhibit a lower activation of the PHC compared with healthy controls as well as an association between PHC volume and ruminative tendencies. Furthermore, previous work from our group (Ambrosi et al., 2019) reported higher connectivity between the PHC and the right Hb in patients with depression and suicidal behavior. Changes in the PHC have also been shown to have a role in the antidepressant response. In particular, in an fMRI experiment in depressed elderly patients (Karim et al., 2018), activation of the PHC was associated with treatment remission to venlafaxine, a serotonin-norepinephrine reuptake inhibitor, while higher metabolism in this area was observed after infusion with ketamine in a PET study in TRD patients (Carlson et al., 2013). Conversely, in the latter, clinical improvement was negatively associated with increased metabolism in the PHC.

Relevant to PHC neurocircuitry is the role of ketamine in the activation of pathways that regulate synaptic plasticity and dendritic growth. In a recent investigation, ketamine was associated with a reversal of depressive-like behaviors and an increase in dendritic spine formation in the PFC of mice as early as 24 hours post infusion (Moda-Sava et al., 2019). Brain-derived neurotrophic factor (BDNF), which is the most abundant neurotrophin in the human brain, has been shown to be necessary for ketamine’s antidepressant action (Chen et al., 2006; Liu et al., 2012). Although BDNF is primarily expressed in the hippocampus and PFC, patients expressing the BDNF Val66Met single nucleotide polymorphism, which is associated with deficits in BDNF activity, have shown lower metabolic activity and FC in the PHC (Wei et al., 2017). Thus, it could be proposed that ketamine-induced neuroplasticity changes in the PFC and hippocampus may at least partially underlie the increased FC we observed between the right Hb and the PHC.

### Limitations

There were several limitations in this study. First, the small size of the Hb makes it difficult to study using functional imaging due to limits of resolution. As mentioned earlier, the clusters we identified were small; therefore, these results should be interpreted with caution. It is especially difficult to distinguish between lateral and medial components, which are believed to have different functions and thus may contribute a source of noise when averaging over the entire Hb. We are also not able to completely rule out possible contamination from adjacent thalamic areas or ventricles, and therefore our results may be underpowered compared with other studies of depression or ketamine. More anatomically specific approaches to delineate the Hb should be implemented in future studies as well as an emphasis on higher-resolution functional imaging based on modern acceleration strategies (such as simultaneous multi-slice acceleration), which were not available at both sites at the time of the acquisition. Second, there was a lack of consistency in the results obtained with the QIDS-SR and MADRS. This could be attributed to the fact that depression is a very heterogenous construct involving abnormalities of multiple brain regions; therefore, observing similar results across scales is not always possible. Also, the QIDS-SR includes more questions aimed to assess vegetative functions, such as sleep quality, appetite/changes in weight, and psychomotor activity compared with the MADRS. While speculative, the associations between improvement of depression, as measured with the QIDS-SR, and increased connectivity between the Hb and occipital and temporal areas could be related to the role of these areas in mediating sleep disturbances in depression (Cheng et al., 2018). Third, the current study did not include a placebo treatment or non-MDD control group; therefore, the effects related to time on the observed changes in the ketamine group cannot be fully assessed. Future studies should consider enrollment of non-MDD healthy controls to evaluate effects at baseline and after treatment. In addition, while our ketamine group sample is larger than existing ketamine neuroimaging studies in depression (n approximately 10–20) (Ionescu et al., 2018), it is smaller than other neuroimaging studies in MDD. Fourth, the parent studies from which the imaging and behavioral data were extracted were not specifically designed to interrogate Hb connectivity. Specifically, each site had different acquisition parameters. Two-sample *t* tests between site 1 and site 2 revealed significant differences in average connectivity in clusters located in the thalamus and the vermis of the cerebellum. However, we believe these differences do not affect the overall interpretation of the data, given that the clusters are outside the areas with significant treatment effects. We also incorporated site as a covariate of non-interest in the analysis. Fifth, we failed to find effects in regions that are known to be anatomically connected to the Hb, particularly the VTA, which could be explained by the small size of these structures and the low spatial resolution of the data. Finally, it is possible, although we believe unlikely, that the differences observed are associated with the repetition of the MRI instead of the effects of ketamine. Additional controlled studies are necessary to explore that possibility.

## Conclusion

In summary, our results show that ketamine’s rapid antidepressant effects were associated with changes in Hb connectivity to brain structures important in mood regulation and provide preliminary clinical evidence of the involvement of the Hb in ketamine’s mechanism of action.

## Statement of Interest

Dr Mathew is supported by the use of facilities and resources at the Michael E. DeBakey VA Medical Center, Houston, Texas. He has served as a consultant to Allergan, Alkermes, Clexio Biosciences, Greenwich Biosciences, Intra-Cellular Therapies, Janssen, Perception Neurosciences, Relmada Therapeutics, Seelos Therapeutics, and Signant Health. He has received research support from Biohaven and VistaGen Therapeutics. Dr Salas is supported by the use of facilities and resources at the Michael E. DeBakey VA Medical Center, Houston, Texas. In the past 5 years, Dr Murrough has provided consultation services and/or served on advisory boards for Allergan, Boehreinger Ingelheim, Clexio Biosciences, Fortress Biotech, FSV7, Global Medical Education (GME), Impel Neuropharma, Janssen Research and Development, Medavante-Prophase, Novartis, Otsuka, and Sage Therapeutics. In the past 12 months, Dr Murrough has provided consultation services and/or served on advisory boards for Boehreinger Ingelheim, Clexio Biosciences, Global Medical Education (GME), and Otsuka. Dr Murrough is named on a patent pending for neuropeptide Y as a treatment for mood and anxiety disorders and on a patent pending for the use of ezogabine and other KCNQ channel openers to treat depression and related conditions. The Icahn School of Medicine (employer of Dr Murrough) is named on a patent and has entered into a licensing agreement and will receive payments related to the use of ketamine or esketamine for the treatment of depression. The Icahn School of Medicine is also named on a patent related to the use of ketamine for the treatment of PTSD. Dr Murrough is not named on these patents and will not receive any payments.
